# Supplementary figures and images for: Living with a left ventricular assist device: Capturing recipients experiences using group concept mapping software
Source: PLoS One. 2022 Sep 21;17(9):e0273108. doi: 10.1371/journal.pone.0273108 (PMC9491568; doi:10.1371/journal.pone.0273108)

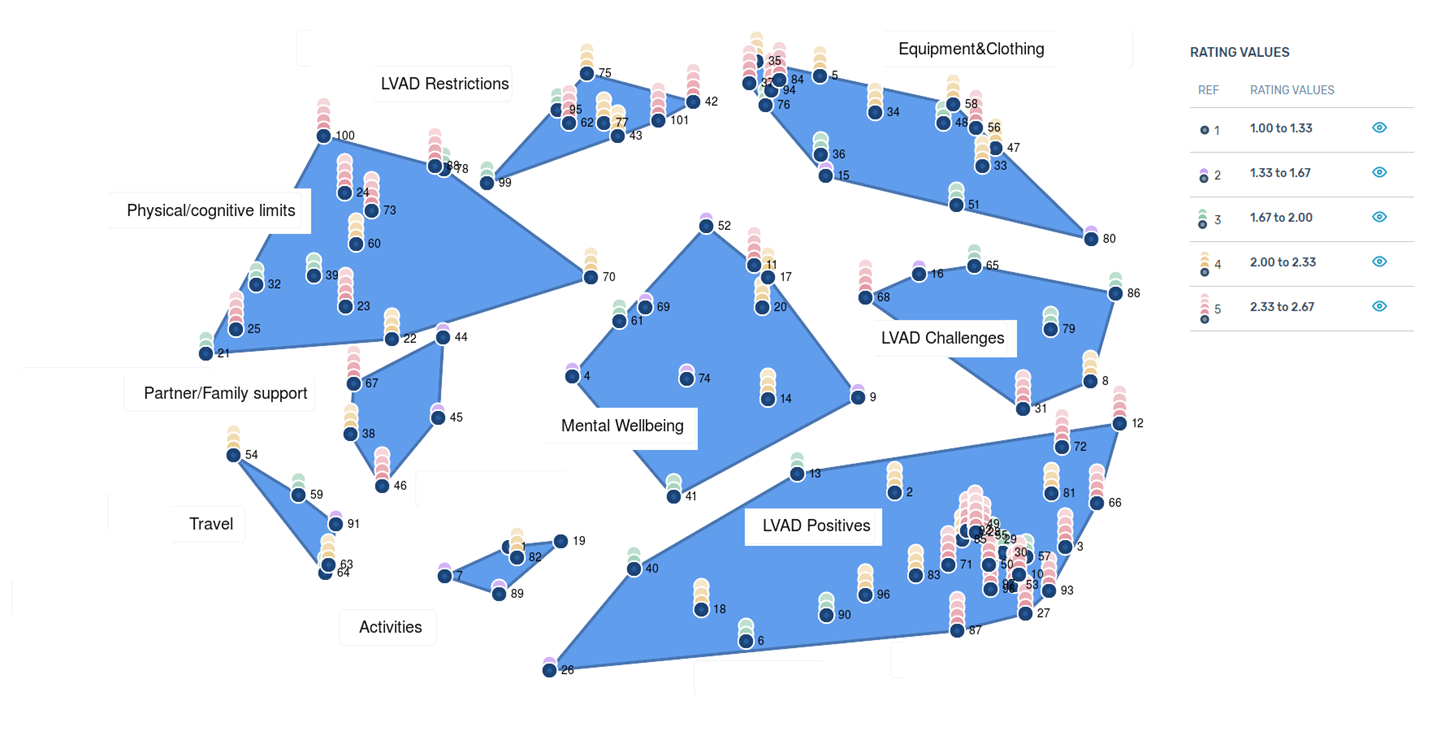

Supplement: S1 Fig — Points map showing which statements were considered important by LVAD recipients. Scoring Importance: 1 = Not important; 2 = Important; 3 = Very important. (TIF) [file pone.0273108.s001.tif]

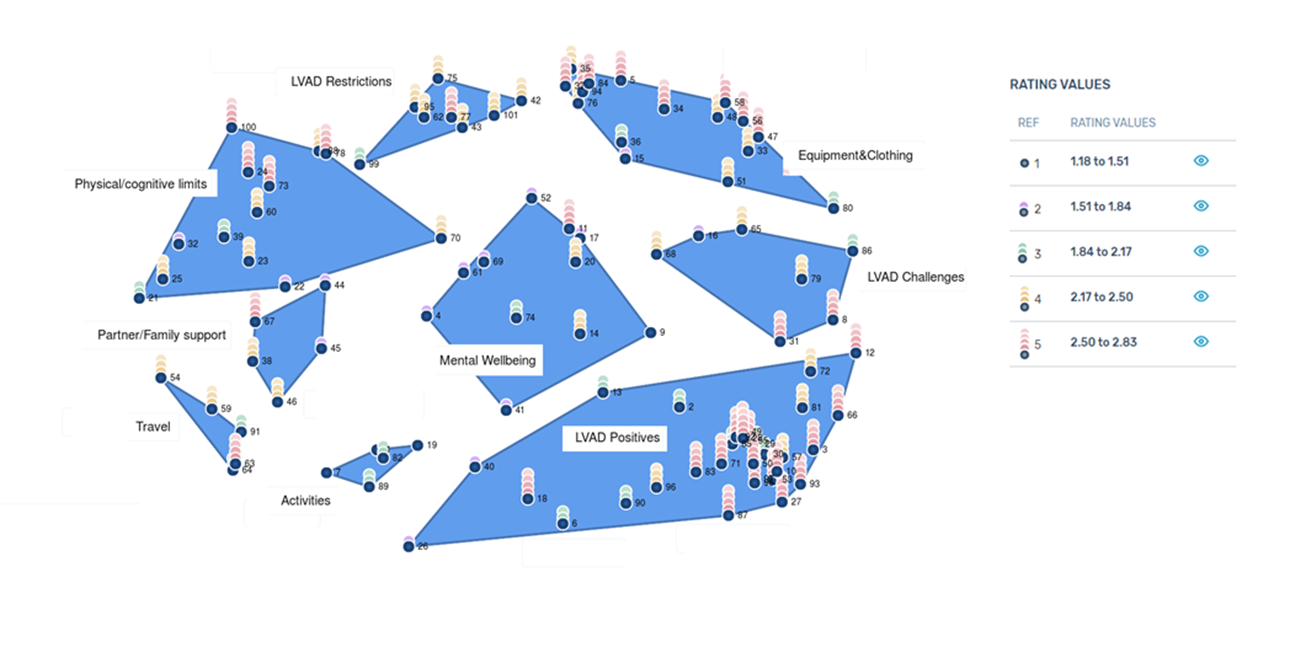

Supplement: S2 Fig — Points map showing which statements most reflected recipients’ experiences. Scoring for relevance: 1 = Not at all; 2 = Sometimes my experience; 3 = Definitely my experience. (TIF) [file pone.0273108.s002.tif]

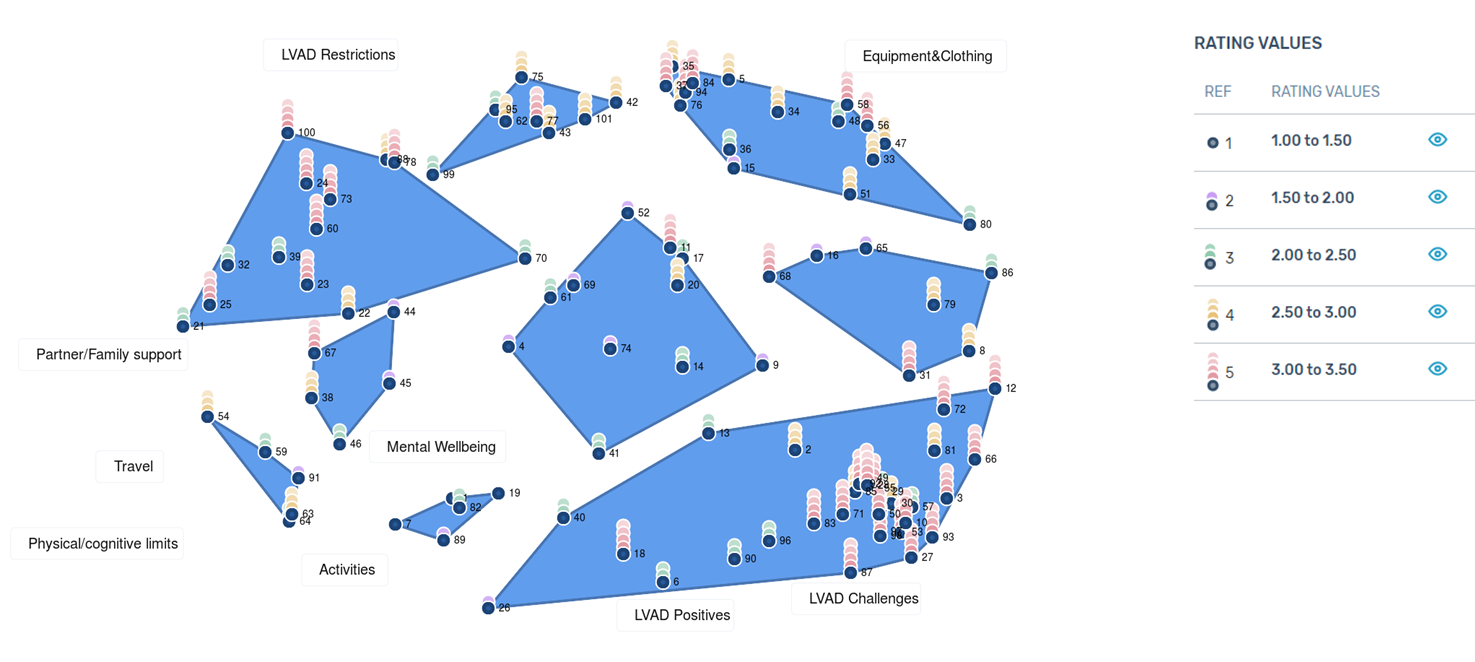

Supplement: S3 Fig — Points map showing which statements recipients thought frequently reflected their experiences. Scoring for Frequency: 1 = Never; 2 = Sometimes; 3 = Frequently; 4 = All of the time. (TIF) [file pone.0273108.s003.tif]
