# Supplementary material for: Living with a left ventricular assist device: Capturing recipients experiences using group concept mapping software
Source: PLoS One. 2022 Sep 21;17(9):e0273108. doi: 10.1371/journal.pone.0273108 (PMC9491568; doi:10.1371/journal.pone.0273108)
Supplement: S1 Protocol — (PDF) [file pone.0273108.s005.pdf]

GCM Protocol

LIVING WITH AN LVAD: DEVELOPING A CONCEPTUAL FRAMEWORK USING GROUP CONCEPT MAPPING

CONFIDENTIAL

LIVING WITH AN LVAD: Developing a conceptual framework using group concept mapping

Aims: To develop a conceptual framework on health-related quality of life for LVAD recipients using group concept mapping.

Contents

PROJECT SUMMARY iv
ABBREVIATIONS vi
KEY WORDS vi
1. Background to the project 7
1.1 Recipients perspectives on living with an LVAD 7
1.2 Rationale for project 7
2. Theoretical framework 8
3. Research question and aims 9
3.1 Aim 9
4. Methods 9
4.1 Recruitment 9
4.2 Phase 1: Development of a Concept Elicitation Framework 10
4.3 Conceptual Framework 10
4.4 Group Concept Mapping 10
4.5 GCM Method 12
5. PPI involvement in the project 12
6. Project setting 13
6.1 Sample and recruitment 13
6.1.3 Eligibility Criteria 14
Data Collection 14
7. Data Analysis 15
7.2 Data Analysis 15
8. Ethical and regulatory considerations 16
8.1 Potential Benefit for participants 16
8.2 Risks and Burdens for participants 16
8.3 Risks to the Researcher 17
8.4 Research Ethics Committee (REC) and other Regulatory review & reports 17
8.5 Regulatory Review & Compliance 17
8.6 Peer review 18
9. Patient & Public Involvement 19
10. Data protection and patient confidentiality 19
11. References 20

PROJECT SUMMARY

Advanced heart failure (AHF) carries a high mortality rate, with heart transplantation offering good outcomes for patients. Due to the limited donation rates waiting list mortality is high. A left ventricular assist device (LVAD) is an alternative complimentary therapy for AHF. The pump supports the systemic circulation making it less reliant on the failing left ventricle, allowing patients to mobilise, exercise and return home or even return to full time work.

A patient participation initiative (PPI) involving LVAD recipients demonstrated that there were a number of issues and concerns related to living with an LVAD that were not being addressed by currently available questionnaires. This group suggested that a new tool in the form of a patient reported outcome measure (PROM) was required to assess the impact of living with an LVAD on their health-related quality of life (HRQoL). PROMs are self-completed questionnaires that evaluate a patient's health from their perspective, and should reflect the issues that are important to specific clinical populations.

Previous studies, have demonstrated that PROMs developed with input from people who have experience of living with the health condition are more sensitive and have superior content validity.(1-3) The European Medical Agency (EMA) and Food and Drugs Agency (FDA) guidelines (2, 3) advocate that identification or development of PROMs for use in clinical trials should be underpinned by a conceptual framework with patients contributing to that framework.(4, 5)

A systematic review found no PROMs specifically developed with LVAD patient input for use in the UK and few studies of PROMS addressing psychological issues in LVAD recipients suggesting that a better HRQoL tool capturing all domains relevant to LVAD recipients is required and this should be underpinned by a conceptual framework. As part of a larger project group concept mapping will identify key domains and items that are important to recipients and contribute to a conceptual framework..

**Project Title**	Living with an LVAD: Developing a conceptual framework using group concept mapping
**Project Design**	Development of a conceptual framework using using Group concept mapping (GCM),
**Project Participants**	Recipients or previous recipients with experience of living with a Left Ventricular Assist Device
**Planned Size of Sample (if applicable)**	Group Concept Mapping n=15 to 20
**Planned Project Period**	1 April 2019 to 31 January 2023
**Research Question/Aim(s)**	Development of a conceptual framework with input from current or previous LVAD recipients. As part of a larger project to develop a PROM which reflects recipients' experiences of life with an LVAD using group concept mapping.

ABBREVIATIONS

Advanced heart failure (AHF)

Birmingham Cardiopulmonary Transplant Service (BCTS)

European Medical Agency (EMA)

Food and Drugs Agency (FDA)

Group concept mapping (GCM)

Health Related Quality of Life (HRQoL)

Kansas City Cardiomyopathy Questionnaire (KCCQ),

Left Ventricle (LV),

Left Ventricular Assist Device (LVAD)

LVAD patient advisory group (LPAG)

Minnesota Living with Heart Failure Questionnaire (MLHFQ)

Patient Participation Initiative (PPI)

Patient Reported Outcome Measures (PROMs)

KEY WORDS

Left Ventricular Assist Device, Patient Reported Outcome Measures (PROMs), Health Related Quality of Life (HRQoL), Cardiac Surgery, Heart Transplantation.

PROJECT PROTOCOL

1. Background to the project

Advanced Heart failure (AHF) is a complex clinical syndrome defined as the heart's inadequacy to pump blood to the end organs it supplies. LVADs are implantable mechanical support pumps sustaining circulation in AHF patients. LVAD pumps support the left ventricle (LV), by providing an alternative route for the blood. Implanted within the pericardium, an inflow cannula drains blood from the LV to the pump, driving blood through an outflow cannula to the aorta. Electrical and informatics cabling is encased within the drive-line which is subcutaneously tunnelled to an external computerised controller and electrical supply (mains or portable batteries weighing about 2 kg).

1.1 Recipients perspectives on living with an LVAD

Despite health and AHF symptom improvements, receiving an LVAD brings its own issues, and requires substantial environmental and lifestyle changes for recipients and their families. In an attempt to understand these changes, eight recipients (one female and seven males) participated in a public and patient involvement (PPI) group meeting. One patient had received an LVAD within the previous month, three had subsequently received a transplant, the length of time that the other members had been living with an LVAD ranged for 1 to 6 years.

The group identified a wide range of psychological and practical issues related to living with the LVAD. Living with an LVAD affected all aspects of their life, requiring major physical, social, and environmental adaptation. They reported both positive and negative psychological consequences on them, and their relationships as a result of receiving an LVAD.

1.2 Rationale for project

For PROMs to be effective in clinical trials and practice, they have to capture information on domains that matter to the patient.(4)

Our PPI group evaluated some frequently used PROMs, including the Kansas City Cardiomyopathy Questionnaire (KCCQ), Minnesota Living with Heart Failure Questionnaire (MLHFQ), and the SF-36 a generic quality-of-life measure, frequently used across a wide range of patient groups. The PPI group felt that many of the symptoms captured by the heart-specific measures were no longer relevant, and the SF-36 did not address some of the unique, specific and problematic aspects of their lives. They expressed a wish for a PROM that reflected their unique experiences across all domains including physical, social, emotional, and practical functioning.

CONFIDENTIAL

This project aims to gain a wider understanding of the issues that are important to LVAD recipients and develop a conceptual framework, as part of a larger project to develop a PROM for use in research and routine clinical practice.

2. Theoretical framework

The European Medical Agency (EMA) and Food and Drugs Agency (FDA) guidelines, advocate that PROM development and selection should be supported by a conceptual mapping framework and patients should contribute to that framework.(2, 6, 7) A conceptual framework based on a group conceptual mapping exercise will capture the breadth of issues that LVAD recipients experience as a result of LVAD implantation.(4, 8)

CONFIDENTIAL

3. Research question and aims

3.1 Aim

This project aims to explore the impact of living with an LVAD on recipient's quality of life, and develop a conceptual framework as part of an ongoing project to develop a bespoke measure of health-related quality of life with input from current and previous LVAD recipients.

Objectives

- To identify the lived experiences of LVAD recipients and the impact of living with an LVAD on health-related quality of life
- Identify the range of issues, domains and items that are important to LVAD recipients for inclusion in a future PROM

4. Methods

4.1 Recruitment

Potential participants will be recruited through the LVAD co-ordinators at Birmingham cardiopulmonary transplant service (BCTS), they have regular contact with LVAD recipients. During their routine contact with the LVAD co-ordinator, patients will be asked if they would be interested in the project, and if they would like to be sent an invitation letter, and a consent to contact form. If they agree they will be sent a consent to contact form which they can return to the research team either electronically or by post. Upon receipt of the completed consent to contact form, the researcher will send out the full information pack containing the patient information sheet, demographic questionnaire and consent form. Potential participants will be enrolled into the study once the research team have received their consent. Returning the consent form and demographic questionnaire maybe done electronically if patients opt to receive information electronically, or by hard copy if participants have opted to receive information by post (a pre-paid envelope will be supplied). Participants will be sent a reminder email or letter at a minimum 2 weeks after the initial information pack was sent to them.

Patients who previously had an LVAD and have gone on to be transplanted or explanted are also known to the coordinators and some are still in regular contact for monitoring. They will also be contacted by the coordinator who will ask them if they are interested in the project if they say yes, they will be sent a consent to contact form which they can return to the research team electronically or by post. The researcher will then send them, copies of the participant information sheets (PIS), a demographic questionnaire and a consent form, either electronically or in hard copy if that is their preference. They can then return a copy of the consent form and demographic questionnaire to the researcher. Patients

CONFIDENTIAL

opting to receive information by hard copy can return the information in the pre-paid envelope supplied. They can also contact the researcher if they have any questions as contact details are on the consent to contact form and the other information e.g. PIS. They will also be sent a reminder email or letter at a minimum 2 weeks after the initial information pack was sent to them using their preferred contact method.

4.2 Phase 1: Development of a Concept Elicitation Framework

There have been several guidelines written on developing PROMs and the FDA expects new PROMs being used to support labelling claims to have gone through this process.(2, 7, 9) These guidelines have been used to develop the overall project design.

4.3 Conceptual Framework

A conceptual framework based on a group conceptual mapping exercise will help establish which items, domains and general concepts are important to LVAD recipients for evaluation in a PROM.(2, 4, 6, 9). This project relates to the group concept mapping exercise of the project.

4.4 Group Concept Mapping

Group concept mapping (GCM) is a relatively new method for evaluating and developing PROMs.(10) Primarily developed as a social science research process, it gives stakeholders a voice, and opportunity to have their views heard. In health, GCM is a useful tool for identifying the issues that are important to patients living with a particular health condition, and the impact it has on their life. GCM can generate a range of ideas structured around patients' choices, and grouped according to their own experiences of living with a health condition. GCM not only illustrates which symptoms are important, but how much of an impact the symptoms have on their daily life. GCM can also identify relationships between ranges of symptoms and different domains such as self-care. Cluster analysis can produce graphical representations of similarities and inter-relationships between domains based on patients' experiences.(9-11) The identification of clustered sets of common symptoms, experiences and their severity can provide a practical method of mapping statements from the GCM exercises and qualitative interviews against potential domains identified by the cluster analysis and psychometric analysis.(9, 10)

CONFIDENTIAL

Figure 1. Group Concept Mapping

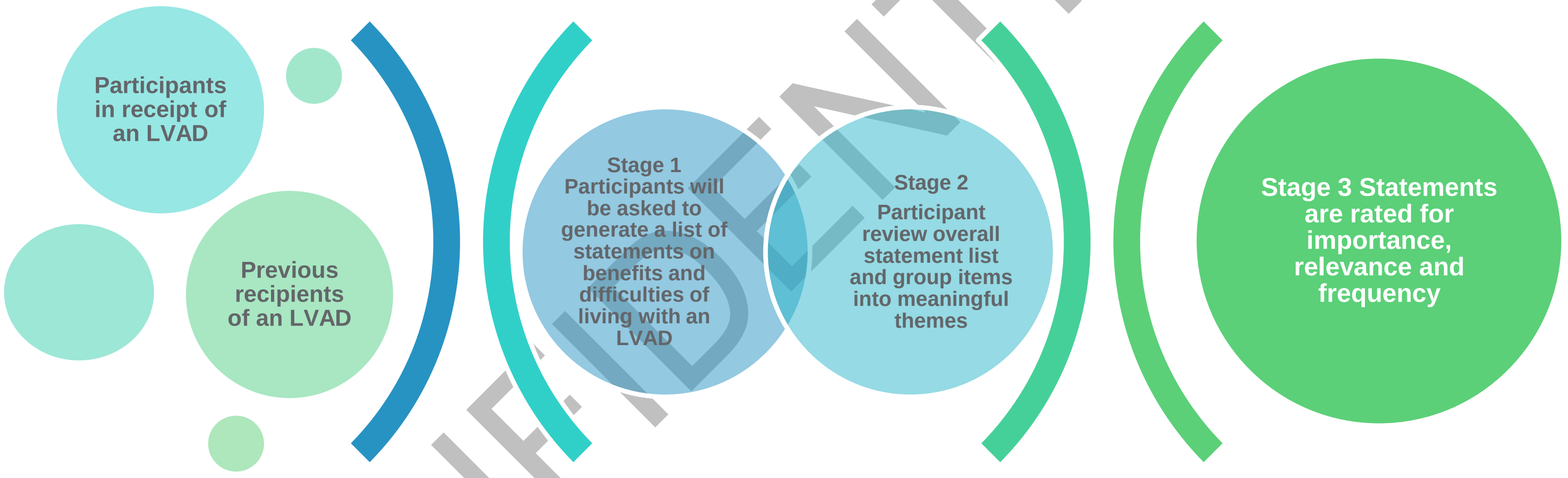

Participants in receipt, explanted or transplanted after receiving an LVAD will be contacted via an LVAD co-ordinator

Exercises 1 and 2

Each individual generates a statement relating to impacts and symptoms

Cluster analysis to identify key domains and potential areas for important domains and items

CONFIDENTIAL

4.4.1 Reasons for using GCM

GCM would allow us to gain a broader view of the issues impacting on LVAD recipient's quality of life; this would provide information that could be explored in depth using qualitative interviews. Given the small number of LVAD recipients and the range of age groups and geographical locations of potential participants this will allow us to gain a broader perspective of their issues.

4.5 GCM Method

Group concept mapping (GCM) will be carried out online using a GCM mapping software GLOBALMAX™ software. This consists of three stages 1) Recipients generate statements in response to a prompt on the many impacts or symptoms that affect them as a result of having an LVAD, 2) Duplicate items are removed and a final list is generated from the information supplied by recipients. Recipients are asked to do a second exercise where they group statements from the amalgamated list in a way that is meaningful to them, 3) Rate statements for importance, relevance and frequency. Participants who opt to do the GCM exercise will be sent a link to the website to generate their list. When everyone has completed exercise 1 participants will be sent additional links for the other stages. Cluster analysis will be used to identify potential items and domains for the conceptual framework. Participants who have consented to do the tasks but not completed task within 2 weeks of being sent links will be sent a reminder letter or email.

5. PPI involvement in the project

Patient partner involvement has underpinned the development of this research project throughout, it was through PPI consultations that we found that despite health and AHF symptom improvements there were some HRQoL concerns. LVAD recipients discussed how receiving an LVAD created its own issues and required substantial environmental and lifestyle changes for recipients and their families. They reported both positive and negative psychological consequences for them and their relationships.

An important part of this project is the continued involvement of patient partners and the LVAD patient advisory group (LPAG), which includes five of the original PPI group, will continue to collaborate with the research team throughout the project. Some of the other original PPI members have opted to keep in touch by email. These members, and LPAG have contributed to the design of the project and reviewed documents such as consent forms and information sheets. One of the PPI group was CI on the research grant, and is currently part of the project group and continues to be a member of LPAG. In line with current INVOLVE PPI guidelines, LPAG have contributed to the design of documentation for the project, e.g. consent forms and patient information sheets, and have made suggestions for patient recruitment and design of the project. They will be attending meetings twice a year to support the project and this will include a range of activities including reviewing data analysis, and drafts of the PROM items,

CONFIDENTIAL

procedures for recruitment and trialling web-based survey/paper and pencil formats as well as being involved in dissemination of the final results. LPAG members will be reimbursed travel and subsistence expenses for attending the meetings for them and any carers travelling with them.

6. Project setting

6.1 Sample and recruitment

6.1.1 Birmingham Cardiopulmonary Transplant Service

Birmingham is one of the leading centres for LVAD surgery with a catchment area which covers Wales and the South West through to London and the Midlands.

6.1.2 Recruitment

Participants will be recruited through the BCTS. The BCTS LVAD co-ordinator will act as a gatekeeper and identify potential patients using the inclusion and exclusion criteria (as patients are well known to the coordinator). During routine clinical calls to patients which is part of their ongoing care (often weekly), the coordinator will ask them if they are interested in the project and whether they would like to be sent information on the project. If they express an interest in the project, they will be sent an invitation and a copy of the consent to contact form. This may be sent to them electronically or in hard copy if they prefer. They will return the consent to contact form to the research team either electronically or by post. The researcher will then send copies of the participant information sheet, a basic demographic questionnaire and consent form to them. They will be able to say on the consent form which stages of the research they wish to take part in, and return it to the researcher with their demographic questionnaire within two weeks of receiving the information sheet. They will also be able to contact the researcher if they would like further information or have any questions. A reminder will be sent at a minimum 2 weeks after they were sent the information.

Patients who have been transplanted or explanted are also known to the co-ordinator, and mostly still in regular contact with the coordinator. The LVAD coordinator will identify potential participants who fit the inclusion criteria and contact them to see if they are interested in receiving information on the study. As above, if they express an interest they will be sent an invitation and consent to contact form and asked to sign the consent to contact form and return it to the research team. They will be sent the PIS, consent form and demographic questionnaire either electronically or as a hard copy depending on their preference. A pre-paid envelope will be included for participants opting for hard copies. They will be asked to return the consent form and demographic questionnaire within two weeks of receiving the information if they wish to take part. A reminder will be sent at a minimum 2 weeks after they were sent the information.

CONFIDENTIAL

6.1.3 Eligibility Criteria

6.1.4 Inclusion criteria

All patients over 18 who currently have or previous experience of living with an LVAD will be eligible to take part in the project.

Data Collection

6.1.5 Group Concept Mapping

Stage 1 Concept Generation

An online web-page will be hosted by GlobalMax™ participants will be able to generate a list of statement that reflect their own personal experiences.(11-13). Participants will be given a unique ID and link to the webpage so that they can log into the webpage.

They will be given a prompt relating to their symptoms and impact of living with an LVAD, and asked to generate as many responses as they wish relating to that topic.(11, 12) If participants become tired they will be able to save their lists and come back and complete it at another time.

Participants will be given two weeks to complete the list and a reminder will be sent out two weeks and four weeks after they receive the link if they have not completed Phase 1. At this stage if they decide to withdraw, it will be possible to delete their data if they have completed any of the exercises. Phase 1 will result in a patient-authored symptom and impact list that is representative of their particular experiences of living with an LVAD in their own words.

Stages 2 and 3 Sorting and Rating

The lists for all participants will be consolidated and duplicates removed to create a final list of symptoms and impacts of living with an LVAD.

Sorting

Each participant will then be contacted to complete the second stage of the exercise, which includes sorting the symptoms or impacts into groups of their own design and labelling them in a way that is meaningful to them e.g. social activities, anxiety about the equipment, etc. They can drag and drop all of the symptom impact descriptors into that group e.g. it might consist of issues with the LVAD equipment or physical symptoms.(11, 14, 15)

Rating

Thirdly, they will then be able to say how important the statement is, how often it causes a problem, and how much it reflects their experience. Categories will be split into 0 to 4 groups and defined using appropriate descriptors.(11, 14, 15)

CONFIDENTIAL

6.1.6 Withdrawal

It will be made clear in all of the information sheets that participants can withdraw at any time and this will not have any negative consequences for their treatment or future involvement in clinical trials for patients, or employment (if a staff participant).

During stage 1 it will be possible to delete their data but when data have been amalgamated it will not be possible to identify their information. Data from stage 2 can be deleted while the phase is open, but once it has finished and data have been amalgamated it will not be possible to identify and delete individual data. Data from stage 3 could be deleted until all the data are amalgamated for analysis.

6.1.7 Identification

A member of the research team will generate the unique ID number using Microsoft Excel Files, information linked to the ID will be kept in a separate encrypted file to the other data, on a secure university server which is backed up daily. Only the chief investigator or designee and sponsor (if required for quality assurance purposes) may have access to participant's information, confidentiality will be respected at all times. Hard copies of any consent forms or demographic questionnaires will be stored in a locked filing cabinet in a locked office at the Murray Learning Centre, University of Birmingham. Electronic copies of demographic questionnaires or consent forms will be kept in an encrypted file on a secured computer. This data will be stored for 10 years and then destroyed securely. Any computers used for this project are password protected and kept in a secure locked office within a secured building within the University of Birmingham. Laptops used within the university are encrypted and protected by a pin and a password.

7. Data Analysis

7.1.1 Group Concept mapping

There are no sample size requirements for cluster analysis but we hope to recruit 15 to 20 participants for the GCM part of the project to try and gain a representative mix of patient living with an LVAD and their viewpoints.(8) This is the recommended number to achieve reliability.(16)

7.2 Data Analysis

7.2.1 GCM Data Analysis

Data generated through the GCM exercise will be analysed using GlobalMAX™ software (13) and SPSS. Data generated by the sorting exercises will be collated into a similarity matrix. This examines the extent to which symptoms and impact descriptors are grouped together.

CONFIDENTIAL

Data collected from this procedure will be used to generate a conceptual map of the domains and sub-domains and their relationships using cluster analysis. These identified domains can then be used to map groups of items and domains and this matrix can be used to identify conceptual framework and a range of suitable PROM items to map against these concepts. This will help us identify which items from the potential PROM are aligning with the concepts identified by the mapping exercises.(12, 14, 15)

8. Ethical and regulatory considerations

8.1 Potential Benefit for participants

Research has shown that PROMs that reflect the lived experience of patients make more sensitive and reliable measurement tools. By creating a PROM that reflects real experiences of people living with an LVAD, we are more likely to be able to measure meaningful changes in their quality of life and symptoms as a result of clinical trials, LVAD design evolutions and therapeutic interventions. This is especially important given the increasing use of LVADs, increased life expectancy, and for many LVADS are increasingly becoming a destination therapy. Having the right measurement tools to capture the impact of any new therapeutic interventions or design evolutions on quality of life, is important for the future management of recipients of LVADs and long-term clinical research. Developing a conceptual map is the first stage in the development of a PROM.

8.2 Risks and Burdens for participants

8.2.1 Group Concept Mapping

Participants may feel distressed when completing the exercises especially if they are thinking about their symptoms and its impact on their life. Participants will be advised to contact either a member of their clinical team or LVAD coordinator, or the researcher who will signpost them to an appropriate service for support such as the local Patient Advice and Liaison Service (PALS). Participants who are being cared for by a clinical team will be encouraged to contact their clinical lead for further information and support. If the researcher becomes concerned for the wellbeing of the participant as a result of reading their GCM, they will discuss their concerns with the participant, working in partnership to determine the best course of action. With the participant's permission, the researcher may need to consult with a senior member of the project and/or the treating clinician (if a patient) to address these concerns. In exceptional circumstances, the researcher may need to do this without prior permission if they are concerned for the participant's safety. Participants may also get tired completing the web-based exercise but they can stop and come back to it at a later date if they wish.

CONFIDENTIAL

8.2.2 Sensitive issues

Participants may disclose sensitive issues such as sexual activity or relationship issues in the GCM. Other people taking part in the second phase of the exercise will be told they can leave out anything that they do not feel comfortable with from the list. This is one of the strengths of doing GCM, some of the discussion with the PPI group identified issues around anxieties around forming sexual and personal relationships. Therefore, this needs to be reflected in the exercises. As the information will be anonymised it will not be possible for other participants to know who has completed the exercise or said what. Participants will also be told at the beginning of GCM web-based exercise and in the PIS that we are obliged to report any disclosures of criminal activity or disclosures that require further action to the appropriate authorities.

8.3 Risks to the Researcher

Although unlikely, the subject matter being dealt with is emotive, and therefore this could potentially impact on the researcher. The researcher is an experienced clinician supported by an experienced team who will conduct de-briefing if necessary.

Current COVID-19 guidelines mean social distancing is likely to be needed now and for the foreseeable future as this group are vulnerable and will need to self-isolate.

8.4 Research Ethics Committee (REC) and other Regulatory review & reports

Before the start of the project, approval will be sought from an NHS Research Ethics Committee (REC) for the project protocol, informed consent forms, PIS and other relevant documents.

Any substantial amendments that require review by REC will not be implemented until the REC grants a favourable opinion for the project and the approval letter has been received. All correspondence with the REC will be retained. An annual progress report (APR) will be submitted to the REC within 30 days of the anniversary date on which the favourable opinion was given, and annually until the project is declared ended.

The Chief Investigator will notify the REC of the end of the project. If the project is ended prematurely, the Chief Investigator will notify the REC, including the reasons for the premature termination. Within one year after the end of the project, the Chief Investigator will submit a final report with the results, including any publications/abstracts, to the REC.

8.5 Regulatory Review & Compliance

Before the site enrols participants into the project, the Chief Investigator or designee will ensure that appropriate approvals from participating organisations are in place.

CONFIDENTIAL

For any amendment to the project, the Chief Investigator or designee, in agreement with the sponsor will submit information to the appropriate body in order for them to issue approval for the amendment. The Chief Investigator or designee will work with sites (R&D departments at NHS sites as well as the project delivery team) so they can put the necessary arrangements in place to implement the amendment to confirm their support for the project as amended.

8.5.1 Amendments

If the chief investigator wishes to make a substantial amendment to the REC application or the supporting documents, having obtained the sponsor authorisation, the chief investigator will submit a valid notice of amendment to the REC for consideration. Amendments will be notified to the national coordinating function of the UK country where the lead NHS R&D office is based and communicated to the participating organisations (R&D office and local research team) departments of participating sites to assess whether the amendment affects the NHS permission for that site. The amendment history will be tracked to identify the most recent protocol version.

8.5.2 End of Study and Archiving

The end of project will be when the last copy of the PROM has been completed.
It is the responsibility of the PI to ensure all essential trial documentation and source documents (e.g. signed ICFs, Investigator Site Files, participants' hospital notes, copies of CRFs etc.) at their site are securely retained for at least 10 years. Data will be retained at the University of Birmingham and accessible for a minimum of 10 years after the publication of the final report. In accordance with the UK data archive best practice for researchers recommendations.(17) Data will be stored in open standard formats for long-term preservation of data:

- Digital audio: Free Lossless Audio Codec (FLAC) (.flac)
- Textual data: plain text data, ASCII (.txt)

8.6 Peer review

An outline of this proposal was reviewed by two reviewers prior to submission to the BHF. It was also peer reviewed during the application to the British Heart Foundation. A cardiologist and Professor of Rehabilitation Medicine (with experience of PROM development and evaluation including Rasch analysis) have reviewed this protocol. An experienced psychometrician, with experience of PROM development and analysis using Rasch analysis has reviewed the statistical methodology.

CONFIDENTIAL

9. Patient & Public Involvement

This project is being supported by LPAG, one of the original PPI members is a co-investigator on the research grant and steering group. Members of this group have provided feedback on the methods, such as recruitment strategies, and project documents, including the consent form, PIS and demographic questionnaire. LPAG will continue to collaborate with the research team throughout the duration of the project. Their involvement will include helping address any issues as they arise (such as recruitment), establishing a lay dissemination plan and co-writing the lay summary. The contribution of LPAG will be described in the final project report using the GRIPP2 framework.

10. Data protection and patient confidentiality

Hard copies of consent forms and demographic questionnaires will be stored in a secure locked cabinet in a secure locked office at the University of Birmingham. Participant data will be handled and stored according to the General Data Protection Regulation and Data Protection Act (2018) and the Information Security Policy of the University of Birmingham.

All identifiable electronic data including contact details will be held within encrypted files and stored on a secure server which is backed up daily at the University of Birmingham. The computer used for this project is password protected and will be kept in a secure locked office within a secure locked building within the University of Birmingham.

Participants opting to complete the group concept mapping exercises will be given an ID to use online so that they do not have to include personal information. The ID will be generated by the researcher when they return the consent form and will be sent to the participants with the links to the web-based sites. Information relating to the ID will be kept separately to the other documentation.

It is planned to publish results in an open access publication and disseminate relevant findings through a comprehensive dissemination strategy. Participants will be asked if they would like to be sent a summary of project findings at the end of the interview and, if requested, they will be sent the findings summary via their preferred method of communication.

CONFIDENTIAL

11.References

1. Basch E, Geoghegan C, Coons S, et al. Patient-reported outcomes in cancer drug development and US regulatory review: Perspectives from industry, the food and drug administration, and the patient. JAMA Oncology. 2015;1(3):375-9.

2. Patrick DL, Burke LB, Powers JH, Scott JA, Rock EP, Dawisha S, et al. Patient-Reported Outcomes to Support Medical Product Labeling Claims: FDA Perspective. Value Health. 2007;10:S125-S37.

3. U.S. Food & Drug Administration. Use of Real-World Evidence to support Regulatory Decision-Making for Medical Devices: Guidance for Industry and Food and Drug Administration Staff. Silver Birch, MD: Center for Biologics Evaluation and Research (CBER); 2017 31 August 2017. Report No.: 1500012 Contract No.: 1500012.

4. Rothman ML, Beltran P, Cappelleri JC, Lipscomb J, Teschendorf B, the Mayo FDAP-ROCMG. Patient-Reported Outcomes: Conceptual Issues. Value Health. 2007;10:S66-S75.

5. FDA. Guidance for industry. Patient-reported outcome measures: use in medical product development to support labeling claims. Silver Spring, MD: US Department of Health and Human Services, Adminstration FaD; 2009.

6. EMA. Appendix 2 to the guideline on the evaluation of anticancer medicinal products in man The use of patient-reported outcome (PRO) measures in oncology studies. European Medicines Agency Committee for Medicinal Products for Human Use (CHMP); 2016. Contract No.: EMA/CHMP/292464/2014.

7. FDA. Patient reported outcome measures: Use in Medical Product Development to Support Labeling Claims. In: (CDER) CfDEaRFaDA, editor. Silver Spring, MD: US Dept of Health and Human Services Food and Drug Administration; 2009. p. 39.

8. Sjödahl Hammarlund C, Nilsson MH, Hagell P. Measuring outcomes in Parkinson's disease: a multi-perspective concept mapping study. Qual Life Res. 2012;21.

9. Velozo CA, Seel RT, Magasi S, Heinemann AW, Romero S. Improving Measurement Methods in Rehabilitation: Core Concepts and Recommendations for Scale Development. Arch Phys Med Rehabil. 2012;93(8, Supplement):S154-S63.

10. Rosas SR, Ridings JW. The use of concept mapping in measurement development and evaluation: Application and future directions. Evaluation and Program Planning. 2017;60:265-76.

11. Humphrey L, Willgoss T, Trigg A, Meysner S, Kane M, Dickinson S, et al. A comparison of three methods to generate a conceptual understanding of a disease based on the patients' perspective. J Patient Rep Outcomes. 2017;1(1):9.

12. Trochim WM, McLinden D. Introduction to a special issue on concept mapping. Eval Program Plann. 2017;60:166-75.

13. CSI. CS Global MAX Ithaca, NY: Concept Systems Inc.; 2018 [Available from: https://www.conceptsystems.com/home.

14. Kane M, WMK. T, In:ditors. Concept mapping for applied social research. In: Bickman L, Rog D, editors. The sage handbook of applied social research methods. Thousand Oaks, CA: Sage Publications Inc; 2009. p. 435–74.

15. Sjödahl Hammarlund C, Nilsson MH, Idvall M, Rosas SR, Hagell P. Conceptualizing and prioritizing clinical trial outcomes from the perspectives of people with Parkinson's disease versus health care professionals: a concept mapping study. Qual Life Res. 2014;23.

16. Rosas SR, Camphausen LC. The use of concept mapping for scale development and validation in evaluation. Evaluation and Program Planning. 2007;30:125-35.

17. Van den Eynden V, Corti L, Woollard M, Bishop L, Horton L, . Best Practice for Researchers UK Data Archive.,; 2011.

CONFIDENTIAL
