# Supplementary material for: Living with a left ventricular assist device: Capturing recipients experiences using group concept mapping software
Source: PLoS One. 2022 Sep 21;17(9):e0273108. doi: 10.1371/journal.pone.0273108 (PMC9491568; doi:10.1371/journal.pone.0273108)
Supplement: Attachment — Submitted filename: Response to reviewers 1 and 2 14 04 22 PONE-D-21-38323.docx [file pone.0273108.s006.docx]

# **Response to Reviewers’ comments**

## **Reviewer 1.**

We would like to thank the Reviewer for their positive comments.

**Reviewer #1:** The authors present the results of a quite sophisticated study of a small group (18) of LVAD recipients who are being followed at one of the Heart Failure Canters in UK. The study targets eliciting and evaluating personal experience of living with an LVAD using Concept Group Mapping (GCM) approach. The participants were first asked to generate statements reflecting such experience in several categories. Such statements were then grouped, and the participants have rated those in three categories of relevance, frequency, and importance. All responses were then put through quite complex statistical analysis which evaluated several clusters and allowed comparisons between the clusters and inside each cluster using scores generated for such comparisons. The authors claim that this approach offers important advantage to other Patient Related Outcome Measures (PROMs) often used in evaluating outcomes of LVAD implantation or other life-modifying procedures. The main such advantage comes from the fact that the data are generated by allowing the actual recipients of the treatment (such as LVAD) to make statements describing different aspects of their life with the LVAD in their own words rather than answer prepared questions. There is data in the literature that the most accurate information regarding life with the LVAD comes from those who live with the LVAD. In addition, GCM approach has been successfully applied for concept generation and evaluation in the past. It does seem to have allowed the authors to benefit from working with the data generated by a small number of participants. Overall, the findings of the study do contribute some additional information as to is important to the LVAD recipients at which stage of their with the LVAD. However, it is hard to imagine that this information by itself would influence the direction of development of the new generations of LVADs.

### ***Response to reviewer 1.***

## Thank you for your positive review of our paper. We agree that working with this group of LVAD recipients has given us an overview of their experiences of living with an LVAD, and their positive and negative experiences across a range of issues related to living with the LVAD. We agree that this part of the project itself will not influence the development of a new generation of LVADs but we hope some of the issues highlighted in the equipment and clothing section in particular may help us identify potential solutions with further research in the future. This project is also part of a larger project which has included in-depth qualitative interviews using GCM as a guide for semi-structured interviews. This has allowed us to explore the issues related to living with the LVAD in more depth. We hope that future development of patient reported outcome measures that are specific to LVAD recipients based on the GCM and interviews may address some of their concerns as well as supporting research and development of design evolutions.

## **Reviewer 2**

**Reviewer #2:** The implantation of a Left Ventricular Assist Device (LVAD) changes the life of both the patient and their family. Despite clinical improvement in most cases, the user is faced with equipment that imposes restrictions and changes in their image as a result of the surgery and the driveline. Beyond the usual knowledge about LVAD functioning and device-associated complications (such as infections, bleeding, thrombosis, or hemolysis), the attending physician should try to address the patient's feelings about their device. This manuscript does an excellent job of putting the user experience with LVADs at the center of the research, making it possible to identify domains and items that can be used in the development of Patient-Reported Outcome Measures (PROMs) in future research. Using the online Group Concept Mapping (GCM) software to identify these domains and items was also a great success for the authors, as it allowed people using LVADs who lived in different geographic regions to collaborate with the research. In addition to allowing the creation of statements, the GCM allows the research participant to sort and rate them, making it possible to identify what should be prioritized in future PROMS.

**Response to Reviewer 2**

*We would like to thank the Reviewer for their positive comments.*

Despite the good work done by the investigation team, we were able to list some recommendations to improve the understanding of the information generated by the research.

Figures and Tables:
· Figure 1A: Although the figure looks solid, the language of its legend is not clear, making it difficult to understand. I advise you to put some details on the interpretation of the legend as found in Line 209: "Statements frequently grouped together are located proximally, while less frequently grouped 210 statements are distal to each other."

**Response to Reviewer 2**

We have revisited all the figures and recoloured them to help with the differentiation between the different clusters, and added legends into the text as suggested. We have also included additional explanations in Figure 2 which we hope will make it easier to interpret. Unfortunately, because of the way the bridging and anchor statements figures are generated (within the GCM software) it has not been possible to recolour them. We tried to make them clearer using the online suggestions from PLOS one.

Fig 1. Legend

Legend Fig 1a Points maps are visual representations of the two-dimensional solution generated from the similarity matrix created from participants choices in stage 2. Frequently grouped statements are located proximally while less frequently grouped statements are distal to each other. (see page 12)

Figure 1B: This figure represents the clusters well; however, we suggest that each cluster had a different color to facilitate visualization. It’s interesting to add the description about the size of the clusters found between lines 258 - 260.

Legend Fig 1b Cluster maps are visual representations of the hierarchical cluster analysis solution and represent similarities and inter-relationships between the domains and items. Large clusters represent broader concepts, whereas smaller clusters represent more narrowly focused homogenous domains. (see page 12)

Figure 2: This figure is not clear. We suggest detailing a little more about bridging values in the legend (similar between lines 319 and 325). Also, the lines have similar colors, but different thicknesses, so you could be specified what each line thickness is and there are different color points without explanation.

We have included additional explanations in Figure 2 which we hope will make it easier to interpret. Unfortunately, because of the way the bridging and anchor statements figures are generated (within the GCM software) it has not been possible to recolour them. We tried to make them clearer using the online suggestions from PLOS one.

Figure 3: This figure is the most important, because it shows the importance, frequency, and relevance of each cluster. My suggestion is to put each cluster and line with different colors.

**Response to Reviewer 2**

We have revisited all the figures and recoloured them to help with the differentiation between the different clusters, and added legends into the text as suggested.

Figure 2: This figure is not clear. We suggest detailing a little more about bridging values in the legend (similar between lines 319 and 325). Also, the lines have similar colors, but different thicknesses, so you could be specified what each line thickness is and there are different color points without explanation.

**Response to Reviewer 2**

Thank you for your comments we have added some explanation of the different thicknesses and what the different colour points mean to the figure. Additional information has been added to the legend in the manuscript.

Fig 2 legend

Bridging values ranging from 0 to 1 (Fig 2. & Table 1) and indicate the extent to which sorting responses by participants were similar in stage 2. Higher values suggest a statement is more heterogeneous and has links to broader concepts (Fig 2a). While lower values closer to 0 suggest statements were more homogeneous and more frequently sorted together. These statements can be “anchors” for other statements close to them within the cluster (Fig 2b).[28] Spanning Values (Fig 2) represent how frequently statements were sorted together, a thicker line demonstrates that other statements were frequently sorted with the selected statement (100 *‘I have to look after my equipment as my life relies on it’* and 27 ‘*Without my LVAD I wouldn’t be here’*).

Figure 3: This figure is the most important, because it shows the importance, frequency, and relevance of each cluster. My suggestion is to put each cluster and line with different colors.

**Response to Reviewer 2**

Thank you for your comments, this has been recoloured to match the cluster map.
